# Improving Ductility for Composite Beams Reinforced with GFRP Tubes by Using Rebars/Steel Angles

**DOI:** 10.3390/polym14030551

**Published:** 2022-01-29

**Authors:** Jiansong Yuan, Danying Gao, Yin Zhang, Haitang Zhu

**Affiliations:** 1 College of Civil Engineering, Henan University of Engineering, Zhengzhou 451191, China; zhangyin@haue.edu.cn (Y.Z.); htzhu@zzu.edu.cn (H.Z.); 2 School of Water Conservancy Engineering, Zhengzhou University, Zhengzhou 450001, China; gdy@zzu.edu.cn

**Keywords:** ductility, composite beams, GFRP tubes, rebar, steel angles

## Abstract

The ductile behaviour of composite beams reinforced with glass fibre-reinforced polymer (GFRP) pultruded rectangular tubes was investigated in this paper. The composite beams were reinforced with GFRP tubes and different steel products, aiming to improve their ductility by using steel products. The main parameters were the types of the steel reinforcement, namely rebars and steel angles. The flexural behaviour of four beam specimens was tested by using a four-point bending test. The experimental results show that the yield load of the specimens was determined by the steel products and the ultimate load was controlled by the GFRP tubes. Two ductility methods (displacement ductility and energy ductility) were used to evaluate the change of the ductility. Both the methods confirmed that the ductility of the composite beam was improved in varying degrees by using rebars and steel angles. Moreover, the analysis shows that improving the yield load or decreasing the ultimate load of the composite beams contributed to the improvement of the ductility.

## 1. Introduction

The hollow fibre-reinforced polymer (FRP) pultruded profiles (e.g., FRP rectangular tube or FRP circular tube) have been widely adopted as reinforcing devices to strengthen concrete structures [1,2,3,4,5]. Due to their excellent mechanical properties and excellent durability [6,7,8,9,10], FRP tubes can be used as an external strengthening layer for reinforced-concrete (RC) members, and serve as the stay-in-place formwork contributing to the convenient casting of the concrete [11,12,13,14]. The pultruded profiles normally show higher tensile properties because the fibres are aligned in the longitudinal distribution [15,16]. Therefore, the hollow FRP tubes are preferred in the beam members for tension, and many studies have been conducted on the composite beams reinforced with the FRP pultruded tubes [17,18,19].

When the GFRP profile tubes are used to reinforce the composite beams, some issues should be addressed. Firstly, the relatively low elastic modulus of the GFRP profile tubes causes lower bending stiffness and the larger deflection of beam members [17]. Secondly, the smooth surface of the tube leads to weak bond behaviour between the concrete and tubes [18,19,20,21]. Also, the linear elasticity of the FRP materials causes the lack of ductility and brittle failure for the composite beam members [5]. Among the aforementioned issues, the lack of ductility of the composite members could result in brittle failure, thus posing a serious threat to the safety of the structures. As a result, improving the ductile behaviour of the composite beams reinforced by the GFRP tubes has been a big concern.

Rebars or steel sections have been introduced into the FRP composite structures to improve the ductility in previous studies [22,23,24,25]. The beam members reinforced with both FRP and steel reinforcement could show significant inelastic deformation due to the higher ductility of conventional steel products. Several investigations have confirmed the availability of using steel reinforcement to improve the ductility and the energy-dissipating capacity of the members for seismic applications. For example, Kara et al. [26] and Li et al. [27] investigated the flexural behaviour of composite beams reinforced with FRP bars and rebars; Yu et al. [28] conducted a research study on beam members reinforced with CFRP jackets and steel tubes; Hadi et al. [5] tested beam specimens reinforced with the FRP I-section beams and rebars. Apart from the improvement of the ductile response, the steel reinforcement normally contributes to the higher bending stiffness of the beam members due to the higher elastic modulus than that of the FRP composites. Meanwhile, the steel reinforcement is protected well from corrosion by the GFRP tube used outside the beam members [29,30]. Therefore, two kinds of material can complement each other in the composite beams.

The objective of this research was to improve the ductile behaviour of the composite beams reinforced with the FRP pultruded tubes. Different types of steel reinforcement, namely rebars and steel angles, were employed in the proposed composite beams. The flexural behaviour of four beam specimens were investigated by using the four-point bending test. The experimental results were presented and the ductility behaviour was analysed to evaluate the effect of the different types of steel reinforcement.

## 2. Experimental Program

### 2.1. Test Specimens

**Figure 1:**
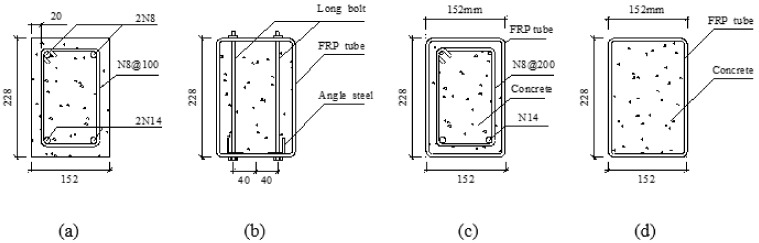
Cross-section of specimens (units: mm): (**a**) RC; (**b**) G-A; (**c**) G-B; (**d**) G-C.

Four beam specimens with different configurations were cast and tested, including one normal reinforced concrete (RC) beam and three composite beams (Figure 1). All the beam specimens had the same dimensions of 3000 mm × 228 mm × 152 mm (Length × Height × Width). The cross-section of the beams was determined by the dimension of the available GFRP tubes. An RC beam was cast as the reference beam and named as RC. In order to improve the ductility of the composite beams, steel angles and steel rebars were used in Specimen G-A and Specimen G-B, respectively. The longitudinal steel reinforcements in Specimens G-A and G-B were designed with the similar tensile yield strength to make sure of the comparability of the beams. The tensile properties of the steel rebars/angles were first tested for the design of the specimens. Based upon the material tests, the dimension of the steel rebars/angles was determined. The diameter of the tensile steel rebars was 14 mm, and the steel angle had a dimension of 30 mm × 30 mm × 3 mm (Length × Thickness). The tensile yield strength of the steel rebars and angles was same in the original design. However, the real yield strength provided by the available materials was not totally same due to the limitations of the industry standard. The deviation of the tensile yield strength provided by two kinds of material was less than 5%. 

For Specimen G-A, the advantages of using steel angles in Specimen G-A was that the steel angles could be fixed at the bottom of the GFRP tubes, as shown in Figure 1b. A total of 12 long bolts were installed at two shear spans (six long bolts were installed at each shear span). The diameter of the long bolts was 6 mm with a length of 250 mm. The six bolts were divided into three pairs, and the three pairs of bolts were installed at three sections of end shear span with a spacing of 300 mm. In this way, the GFRP tubes, steel angles and concrete were connected contributing to a higher shear strength at the interface. Two longitudinal tensile rebars with a diameter of 14 mm were used in Specimen G-B. In order to fix the longitudinal rebars in Specimen G-B, two compressive rebars were placed in the compression zone of the beam, and four stirrups made with steel round bars (8 mm) were evenly distributed along the beam specimen. Specimen G-C is a composite beam reinforced externally with a GFRP pultruded rectangular tube (concrete-filled GFRP tube beam). The details of the specimen information are presented in Table 1.

### 2.2. Material Properties 

GFRP rectangular pultruded tubes with a cross-section of 228 mm × 152 mm × 6 mm (Height × Width × Thickness) were employed, which were fabricated by using the pultrusion process and provided by Nantong Tianmu Insulating Composite Materials Co., Ltd., Nantong, China [31]. The longitudinal tensile strength of the tubes was determined by using ASTM D3039-17 [32]. Five coupons with a dimension of 250 mm × 25 mm × 6 mm (Length × Width × Thickness) were taken from the GFRP tubes, and the measured average longitudinal tensile strength of the GFRP plates was 420.4 MPa. The compressive properties of the GFRP tubes were determined by using ASTM D695-15 [33]. The coupons for the compressive test of the GFRP tubes had a dimension of 125 mm × 25 mm × 6 mm (Length × Width × Thickness) and the measured average compressive strength of the GFRP tubes was 336.5 MPa.

The tensile test on three coupons of the tensile rebars N14 was conducted by using ASTM A370-17 [32]. The measured yield tensile strength and the ultimate tensile strength of the steel bars N14 were 286 MPa and 435 MPa, respectively. The tensile properties of the steel angles were tested by using ASTM A370-17 [34], and the measured yield tensile strength and the ultimate tensile strength of the steel angles were 261 MPa and 392 MPa, respectively. 

Sufficient fluidity is important to ensure the easy casting of the concrete in the tubes. Therefore, self-compacting concrete was employed in this study. The self-compacting concrete was prepared in the laboratory and Table 2 shows the composition. The cubic compressive strength of self-compacting concrete was obtained by using GB/T 50081-02 [35], and three cubes with a dimension of 150 mm × 150 mm × 150 mm were tested. The measured average compressive strength of self-compacting concrete in 28 days was 39.8 MPa.

### 2.3. Fabrication of Specimens 

**Figure 2:**
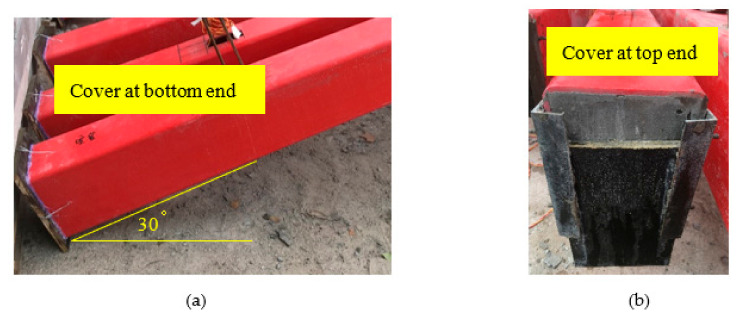
Casting concrete: (**a**) bottom end of specimen; (**b**) top end of specimen.

The RC beam specimen and the CFFT beam specimen had a distinct fabrication process. For Specimen RC, the steel cage was first fabricated and four strain gages were fixed in the middle of the four longitudinal bars. Then, the steel cage was put into the timber formwork prior to the casting of the concrete. The fabrication of the composite beam specimens included the following steps. Firstly, two rectangular holes (100 mm × 50 mm) were drilled in the shear span of the tube to install the hooks, and the hooks were used for the convenient movement of the beam specimens. Then, the steel angles were fixed at the bottom flange of the tube in Specimen G-A, and the prepared steel cages were placed in the FRP tube of Specimen G-B. All the tubes were placed with a slope of 30 degrees for casting concrete as shown in Figure 2a, and the bottom ends of the tubes were covered by using a timber plate. Finally, self-compacting concrete was cast into the three GFRP tubes. The specimens were cured in the ambient environment for 28 days. A single specimen was cast for each configuration due to the large dimension of the beams, and the fabrication and tests of the composite beams were carefully conducted to ensure accurate results.

### 2.4. Test Setup

**Figure 3:**
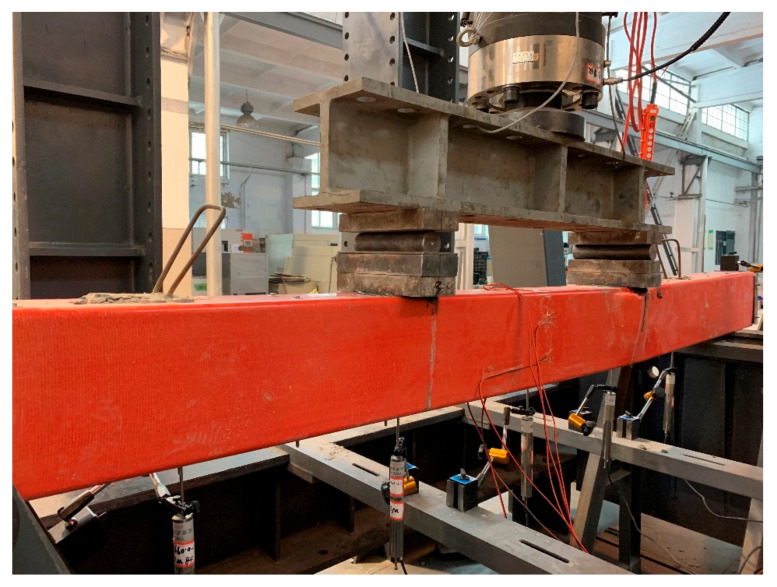
Testing setup.

All the specimens were simply supported and subjected to four-point bending as shown in Figure 3. Each of the specimens had a clear span of 2700 mm. The test beams had a pure bending region of 900 mm and a shear span of 900 mm. Five linear variable differential transformers (LVDTs) were installed to monitor the deflection. The LVDTs, evenly distributed along the beam span, were pointed to the beam bottom flange. The schematic arrangement of LVDTs is shown in Figure 3. Before casting concrete, four strain gages were affixed on the rebars and two on the steel angles. For the GFRP tubes, the strain gages, with two at the top flange, two at the bottom flange and two at the web, were installed on the exterior surface of the GFRP tubes after concrete casting. All the aforementioned strain gages were attached longitudinally at the mid-span section of the beam specimens.

The 200 ton universal testing machine was used to test the beam specimens. The displacement-controlled load with a rate of 0.5 mm/min was applied for loading. The flexural tests were terminated once the ultimate/peak loads of the beam specimens were obtained.

## 3. Experimental Results and Analysis

### 3.1. Failure Modes 

**Figure 4:**
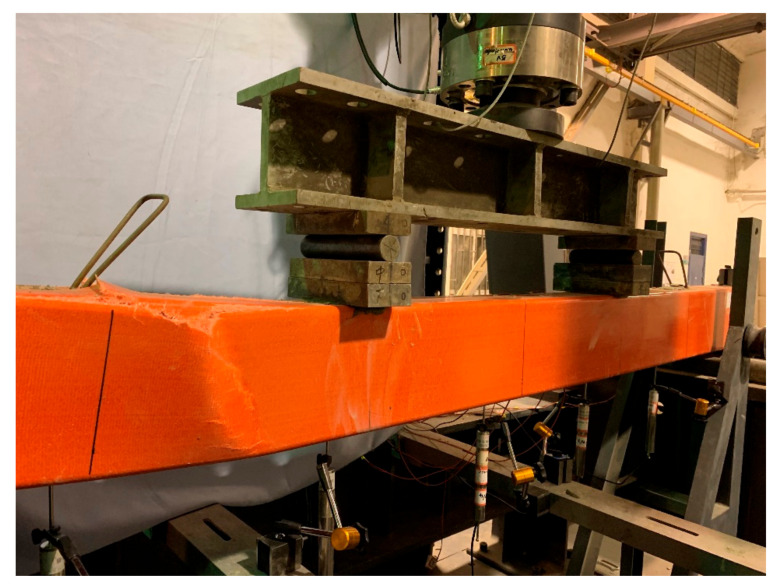
Failure mode.

The composite beams and the RC beam showed different failure modes. Specimen RC failed with the yield of the tensile rebars and crushing of the concrete in the compression zone. For the other three composite specimens, as the load kept increasing, no apparent cracks or damage were found at the GFRP tube before the ultimate load. When the ultimate load was reached, a large explosion was heard and the composite beams failed by the rupture of the top flange nearby one loading point, as shown in Figure 4. This was due to the stress concentration at the top flange caused by the failure of the top flange, and then the composite beam specimens failed. Although the composite beam specimens were damaged, the majority of the GFRP tube was still intact. As a result, the GFRP tubes still possessed high residual strength and elastic deformation. Furthermore, after the load was removed, the deformation of the composite beam specimens was partly recovered. In addition, the concrete core was surrounded by the GFRP tube, and no crushed concrete was found during the tests.

### 3.2. Load-Midspan Deflection Curves

**Figure 5:**
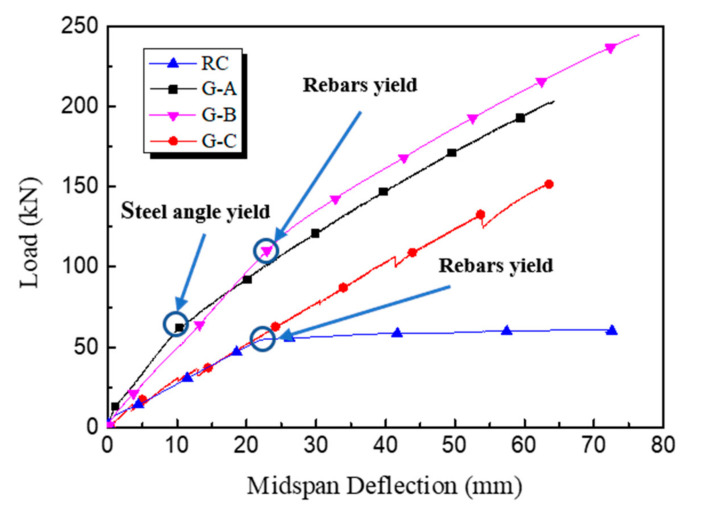
Load–midspan deflection curve.

Figure 5 shows the load–midspan deflection curves of the four beam specimens. Specimen RC performed a typical flexural behaviour of the normal RC beam. At the beginning of the test, a linear increase was demonstrated before the yield point. After the specimen yielded, Specimen RC kept a superior ductile behaviour until the failure (concrete crushing). When the GFRP tube was employed to reinforce the concrete beam in Specimen G-C, the load linearly increased to the ultimate load, and the ultimate load was three times that of Specimen RC. Moreover, several fluctuations were found in the curve of Specimen G-C, which were mainly caused by the cracking of the concrete core. 

The curve of Specimen G-A showed an apparent bilinear increment, and the change of the slope of the load-deflection curve of Specimen G-A was caused by the yield of the steel angles. Before the yield point, the bending stiffness of Specimen G-A was higher compared with that of Specimens G-C and RC. After the yield point, the slope of the load-deflection curve decreased and the curve kept increasing up to the ultimate load. The ultimate load of Specimen G-A improved by 33% in comparison with that of Specimen G-C due to the use of steel angles. For Specimen G-B, the bilinear increment of curves was also observed, while the yield deflection of the specimen was larger than that of Specimen G-A. In addition, the ultimate load of Specimen G-B (250 kN) was the highest among these four specimens, almost five times that of Specimen RC. In general, both the bending stiffness and the ultimate strength of the composite beam specimens were significantly improved when the steel reinforcement was used, however, the composite beam specimen reinforced with rebars exhibited a better flexural behaviour.

### 3.3. Slip between Concrete and Tube

**Figure 6:**
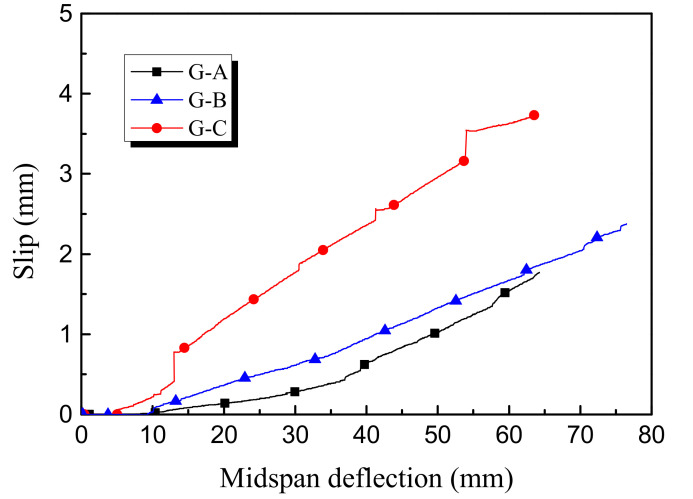
Slip-midspan deflection curve.

Due to the smooth surface of the GFRP tube, the bond between concrete and the tube usually performed poorly, which caused the large slip between concrete and the tube. The average relative slip at the interface was acquired by averaging two LVDTs at two ends of the beam specimens. The slip-midspan deflection curves are given in Figure 6. Specimen G-C shows the largest slip (about 4 mm) among the three specimens, and this is because no measures were taken to control the slip in this specimen. In cases when the rebars and steel angles were employed, the decrease of the relative slip is apparent and the maximum slip is controlled at about 2 mm. The possible reason is that the concrete was well confined by the bond at the interface of the steel reinforcement. When the steel angles were installed in particular, the concrete, GFRP tube and steel angles were connected by long bolts, thus contributing to a superior shear stress at the interface. As a result, Specimen G-A had the minimum slip among the three composite specimens. 

### 3.4. Ductility 

Due to the brittle properties of concrete and GFRP, the composite beams only reinforced with the GFRP tube normally demonstrated brittle failure mode without apparent ductile behaviour. As shown in Figure 5, the weak ductile behaviour of the composite beams is confirmed by the load–midspan curve of Specimen G-C. In cases that the steel angles and the rebars are employed, the ductility of Specimens G-A and G-B improved considerably. Two methods are used to evaluate the improvement of the ductile behaviour as below.

The first one is the traditional displacement ductility method, and the ductile coefficient is computed by using the Equation (1);

(1) $$\mu_{1}=\frac{\Delta_{y}}{\Delta_{u}}$$

where Δ*_u_* is the ultimate midspan deflection, and Δ*_y_* means the yield midspan deflection. Both Specimens G-A and G-B have apparent yield points when the steel angles and the rebars yielded, and Δ*_y_* is obtained at the same time. The ductility coefficient $\left(\mu_{1}\right)$ is given in Table 3. 

The other one is the energy method proposed by Naaman and Jeong [36] as shown in Equation (2), and this method has been verified by several studies [37,38].

(2) $$\mu_{2}=\frac{1}{2}\left(\frac{E_{T}}{E_{E}}+1\right)$$

**Figure 7:**
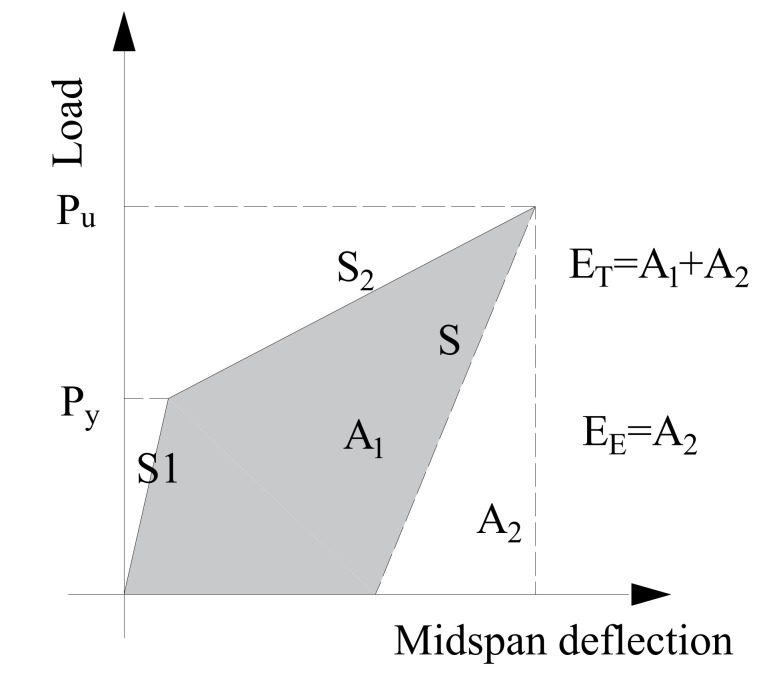
Energy ductility mode.

This method can be employed to analyze the ductile behaviour for the specimens without the identifying yield point, where *E_T_* is the total energy defined as the area under the load–midspan deflection curve of the beam specimens, and *E_E_* is the elastic energy which is calculated as the area under the curve of the elastic stage [39]. The slope of the elastic stage (*S*) is computed by Equation (3):

(3) $$S=\frac{P_{y}S_{1}+\left(P_{u}-P_{y}\right)S_{2}}{P_{u}}$$

where *S*_1_ and *S*_2_ are the slopes in the initial two straight stages of the load–midspan deflection curves. Figure 7 shows the definition of each area, and the energy ductility coefficient ($\mu_{2}$) for the beam specimens is given in Table 3.

### 3.5. Discussion

The ductility coefficient in Table 3 shows that the ductile behavior of the composite beam specimens is improved when using the steel bars and the steel angles. For example, the ductility coefficient of Specimens G-A and G-B is 1.2 and 1.1 by using the energy ductility method, respectively, and the ductility coefficient of Specimen G-C is only 1.0. The ductility coefficient of Specimens G-A, G-B and G-C by the traditional ductility method shows a similar conclusion. Therefore, the improvement of the ductile behavior for the composite beams was validated when the steel products were employed. In addition, two calculation methods showed that using steel angles contributed to a higher improvement of the ductility compared with steel bars. The possible reason may be that the yield strength of the steel angles was lower than that of the steel bars used in this experimental study.

The difference between two calculation methods of the ductility is apparent. Compared with the traditional displacement ductility method, the energy ductility method is more conservative. For example, the ductility coefficient of Specimen G-A is 6.1 by using the traditional displacement ductility method, while the ductility coefficient is only 1.2 by using the energy method. The reason is that the energy ductility method depended on the difference of the flexural stiffness of the beam specimens. However, the difference of the flexural stiffness before and after the yield point (S1 and S2 in Figure 7) was not apparent due to the existence of the FRP tube. As a result, the effect of the steel products on improving the ductility proved to be minor when using the energy ductility.

### 3.6. Ductility Analysis Model

**Figure 8:**
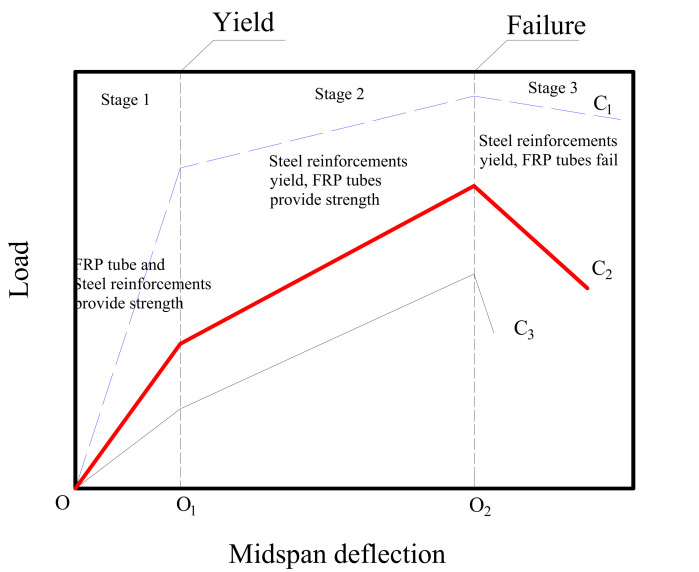
Ductility analysis mode.

According to the measured load–midspan deflection curves of the composite beams reinforced with the steel products and FRP tubes as shown in Figure 5, Figure 8 shows the possible load–midspan deflection curves of the composite beams reinforced with steel reinforcement and FRP tubes. The typical curve C_2_ consists of three stages. At Stage 1, both the steel reinforcement and FRP tube could provide the increasing flexural strength. At Stage 2, the tensile strength of the steel reinforcement remained unchanged. Although the steel products yielded at this stage, the flexural load of the beam specimens still increased due to the contribution of the post-yield strength of the steel reinforcement and the confinement from the GFRP tube. At Stage 3, the GFRP tube failed because the ultimate compressive strain of the GFRP tube was reached. As a result, the load–midspan deflection curve exhibits a sudden drop and the composite beam specimens lose most of the applied load. 

It can be seen from Figure 8 that two points at the curves should be controlled to improve the ductile behaviour of the composite beams reinforced with the GFRP tube and steel reinforcement. The first point is O_1_, where the steel reinforcement experiences yielding. The steel reinforcement yielding contributes to the appearance of the long yield platform at Stage 2. Therefore, the steel reinforcement should be designed with a larger reinforcement ratio, thus improving the yield load of the composite beams. The other point is O_2_, where the GFRP tube experiences failure. The GFRP tube provides a small contribution to the flexural load of the beams, and the difference between the yield load and the ultimate load is small. After the GFRP tube experiences failure at Stage 3, the applied load in the composite beams drops slightly due to the existence of the steel bars, and the sudden brittle failure could be avoided. By increasing the yield load at O_1_ and reducing the ultimate load at O_2_, the load–midspan deflection curve C_1_ is closer to that of the traditional RC beam. In such a way, the ductility of the composite beams is improved. By contrast, if the yield load decreases and the ultimate load increases, the load–midspan deflection curve is closer to the curve of the composite beams only reinforced by FRP composites, such as the curve C_3_ in Figure 8. 

The composite beam specimens in this experimental study performed brittle failure mode. The expected long yield platform at Stage 2 was not found in these composite beam specimens. The reason is that the GFRP tubes possess a high strength but they generally fail in a brittle manner. After the yielding of the steel reinforcement, the applied load kept a significant increase. The difference between the yield load and the ultimate load is large. As a result, the composite beam specimens suddenly lost most of the load carrying capacity once the GFRP tube failed. As mentioned above, when the reinforcement ratio is given, reducing the strength and confinement effects of the GFRP tube is possible in order to improve the effect of the steel reinforcement on raising the ductility of the composite beams. 

## 4. Conclusions

Flexural tests were conducted to investigate the influence of the steel reinforcement on the loading carrying capacity and ductility of composite beam specimens. A total of four specimens were cast, and the test variables included the type of steel reinforcement (rebars and steel angles). The following conclusions can be drawn in accordance with the experimental results and discussions.

The load–midspan deflection curves of the composite beam with the steel angles and the steel bars show an apparent bilinear increment, and the yield of the composite beam was caused by the yield of the steel reinforcements. Both the bending stiffness and the ultimate strength of the composite beam specimens were significantly improved due to the use of the steel reinforcements.

The ductility of the beam specimens was improved by using steel rebars and steel angles. However, the composite beam specimens finally experienced brittle failure mode, and the expected long yield platform was not obtained.

The yield load and the ductile response of the composite beams is controlled by the steel reinforcement. When the yield load of the composite beams is given, a reduction of the ultimate load is beneficial for the improvement of the ductility. The ultimate load of the composite beam specimens is dependent on the GFRP tubes, therefore, a certain reduction of the strength provided by the GFRP tubes is an effective approach to improving the ductile behaviour of composite beams.

This experimental study aims to provide a better understanding of the ductile behaviour of the FRP composite structures. Although the ductile behaviour of the composite beam specimens is improved by steel reinforcement, the brittle failure mode of the specimens is not fundamentally avoided. More detailed parameters should be taken into consideration in future studies based on the analysis in this study, such as the thickness of the GFRP tube, the compressive strength of the concrete, and the different reinforcement ratio of the steel reinforcement.

## Figures and Tables

**Table 1:** Configuration of four specimens.

**No.**	**GFRP Tube (mm)**	**Steel Angles**		**Long Bolts**		**Tensile Steel Rebars**		**Compressive Steel Rebars**		**Stirrups**		
		**Dimension (mm)**	**Number**	**Diameter (mm)**	**Number**	**Diameter (mm)**	**Number**	**Diameter (mm)**	**Number**	**Diameter (mm)**	**Number**	**Spacing (mm)**
RC	-	-	-	-	-	14	2	8	2	8	30	100
G-A	3000 × 228 × 152	30 × 30 × 3	2	6	12	-	-	-	-	-	-	-
G-B	3000 × 228 × 152	-	-	-	-	14	2	8	2	8	4	750
G-C	3000 × 228 × 152	-	-	-	-	-	-	-	-	-	-	-

**Table 2:** Composition of concrete.

Constituent	kg/m^3^
Water	218.9
Cement	357.5
Fly ash	153.2
Coarse aggregate	776.5
Sand	831.6
Water-reducing agent	4.1
Expansive agent	5.1

**Table 3:** Experimental results.

Specimen	Yield Load, *P_y_* (kN)	Yield Midspan Deflection, Δ*_y_* (mm)	Ultimate Load, *P_u_* (kN)	Ultimate Midspan Deflection, Δ*_u_* (mm)	Ultimate Slip, (mm)	$\mathrm{Ductility}\;\mathrm{Coefficient},\;\mu_{1}$	$\mathrm{Ductility}\;\mathrm{Coefficient},\;\mu_{2}$
RC	54.5	22.2	60.0	72.6	-	3.3	3.4
G-A	62.7	10.5	203.1	64.2	1.8	6.1	1.2
G-B	122.1	25.9	244.6	76.5	2.3	3.0	1.1
G-C	-	-	151.8	63.3	3.7	1.0	1.0

## Data Availability

The data presented in this study are available on request from the corresponding author.
